# An approach to scoring cursorial limb proportions in carnivorous dinosaurs and an attempt to account for allometry

**DOI:** 10.1038/srep19828

**Published:** 2016-01-27

**Authors:** W. Scott Persons IV, Philip J. Currie

**Affiliations:** 1 Department of Biological Sciences, University of Alberta, Edmonton, Alberta, Canada

## Abstract

From an initial dataset of 53 theropod species, the general relationship between theropod lower-leg length and body mass is identified. After factoring out this allometric relationship, theropod hindlimb proportions are assessed irrespective of body mass. Cursorial-limb-proportion (CLP) scores derived for each of the considered theropod taxa offer a measure of the extent to which a particular species deviates in favour of higher or lower running speeds. Within the same theropod species, these CLP scores are found to be consistent across multiple adult specimens and across disparate ontogenetic stages. Early theropods are found to have low CLP scores, while the coelurosaurian tyrannosauroids and compsognathids are found to have high CLP scores. Among deinonychosaurs, troodontids have consistently high CLP scores, while many dromaeosaur taxa, including *Velociraptor* and *Deinonychus*, have low CLP scores. This indicates that dromaeosaurs were not, overall, a particularly cursorily adapted group. Comparisons between the CLP scores of *Tyrannosaurus* and specimens referred to the controversial genus *Nanotyrannus* indicate a strong discrepancy in cursorial adaptations, which supports the legitimacy of *Nanotyrannus* and the previous suggestions of ecological partitioning between *Nanotyrannus* and the contemporaneous *Tyrannosaurus*.

Because direct behavioral observations are impossible, assessing the running speeds of fossil taxa is, except when fossil trackways are available^1^,^2^ or when attempting range-bound biomechanical simulations^3^,^4^,^5^, usually limited to the recognition of anatomical traits that are known to correlate with locomotor performance in modern animals. Chief among these traits are limb proportions, most typically: proportional limb length. Other factors being equal, longer limbs impart greater maximum running speeds, because elongated limbs permit greater stride length and effectively allow an animal to cover more ground in a single step^6^,^7^,^8^,^9^,^10^,^11^. Although, during walking and other gaits at slow speeds, short-limbed animals may be able to compensate for limited individual stride lengths by increasing stride frequency, during running, stride length has been shown to be an overwhelmingly important factor influencing maximum speed^6^,^7^,^9^,^10^.

Broadly speaking, femoral length is a hindlimb dimension that varies little with regard to speed, and femoral length seems to be relatively stable in comparison with body length, skull length, ilium length, etc.^11^,^12^,^13^,^14^,^15^,^16^. Assessments of cursorial limb elongation usually consider the length of the lower leg (i.e. from the knee down). Among modern animals, lower-leg length is a statistically significant predictor of relative running speed^10^,^17^, although its predicative power is strongly improved when comparisons are limited to closely related groups with similar overall limb anatomy and locomotive styles^10^.

Non-avian theropod dinosaurs (hereafter referred to simply as “theropods”) include a diverse array of carnivorous forms, but most have hindlimb morphologies that are strikingly conservative^18^,^19^,^20^. This makes theropods a prime group in which to consider cursorial limb proportions. Such considerations have important implications for theropod ecology, because the speeds at which predators can run is a crucial factor that influences their prey choice, hunting strategies, and the habitats in which they are most successful. Previous quantitative studies on the cursorial adaptations of theropods have generally focused on the absolute speeds of particular taxa^3^,^21^,^22^,^23^,^24^. One generally consistent result of most such studies is that the vast range in absolute body size throughout theropods has a strong influence on predictions of speed, because increasing body mass imposes increasing limitations on muscle capabilities and on bone and joint strength^22^,^24^. Proportionate to body mass, more elongate lower legs are typically found among smaller-bodied theropods^11^,^16^,^25^. This general rule holds true in comparisons between both closely and disparately related taxa and throughout the ontogeny of a single species—with smaller juveniles having proportionately more elongate lower-legs than larger adults (Fig. 1)^11^,^16^.

In this paper, we do not estimate absolute or relative maximum running speeds, but rather quantify and compare theropod limb proportions to assess their relative cursoriality^11^,^26^. It is the aim of this study to simply evaluate how strongly the morphology of a particular theropod species has been evolutionarily modified in favor of enhanced cursorial ability, regardless of whether or not the end result of that modification is a maximum running speed that is greater or less than that of other species. In this way, we infer phylogenetic differences in cursoriality, which most logically reflects differential selective pressures for rapid or efficient locomotion. To this end, the allometric effects of body mass on theropod limb proportions is a serious potentially confounding problem. Because allometric restrictions and cursorial adaptive pressures are effectively competing forces that exert opposite influences on the limb morphology of any particular theropod, the one has the potential to obscure the other^11^. For instance, a theropod lineage that is exposed to adaptive pressures favoring cursoriality may show relatively unaltered or even diminished lower limb proportions if, at the same time, that lineage undergoes an increase in body mass and allometric pressures match or exceed selective pressures favoring cursoriality. Similarly, a theropod lineage that undergoes a reduction in absolute body mass may show greater proportional limb elongation, not because the lineage has been exposed to greater selective pressures that favor cursoriality but simply because allometric pressures have been relieved. Carrano^11^ termed this concealing allometric effect on limb proportions “muting” and “enhancing”. This problem has limited large-scale studies of theropod cursorial evolution, and an approach is offered here by which allometric effects on theropod limb proportions can first be identified and then factored out, giving a true measure of the extent to which hindlimb proportions have been modified in favor of cursoriality.

## Material and Methods

### Composition of the initial dataset

Hindlimb length measurements were taken from an initial set of 53 theropod specimens (Table 1). In instances when multiple valid specimens were available for a particular species, only a large specimen was selected, with the aim of restricting the initial dataset to ontogenetically mature individuals. Femur length and metatarsal III length were measured from the proximal-most to the distal-most extent of both bones. It was not possible to identify the distal-most extent of the tibia in all taxa, because the distal end is frequently obscured by fusion with the astragalus and calcaneum. For this reason, “tibia length” for all taxa is actually a measure of the combined proximodistal length of the tibia and the astragalus/calcaneum, when all bones are held in tight articulation. The term “lower leg-length” is here used to refer to the combined proximodistal length of the tibia (plus the astragalus and calcaneum) and metatarsal III. Several previous studies of limb proportions have found that, within the lower leg, it is simply the proportional length of the metatarsals that typically indicates cursorial morphology, and that the length of the tibia is frequently irrelevant. We have chosen to combine both lengths into a single measure in the interest of inclusivity and because tibia length has been recognized as allometrically variable among some theropods^16^.

In the interest of consistency, all measurements were taken directly by one of either of the two authors (not pulled from previously reported data within the literature). All measurements were made directly from the fossils or, in cases when quality was judged equivalent, from casts (no measurements were made indirectly from photographs or illustrations). Standard tape measures were used for large specimens and digital calipers were used for small specimens.

The goal of this study is only to consider limb proportions in carnivorous theropods, and several large, but presumed herbivorous or largely-herbivorous, theropod groups are notably omitted from the initial set of specimens (e.g. “elaphrosaurs”, ornithomimosaurs, oviraptorosaurs, and therizinosaurs). There are three major reasons for not including possible herbivorous taxa. First, the ecological differences between carnivores and herbivores undoubtedly imposes different pressures on locomotor evolution. Cursorial morphology in large herbivores frequently relates more to endurance and the ability to continuously forage across expansive home ranges. Second, many herbivorous theropods have unique foot and limb morphologies. For instance, advanced therizinosauroids have four weight-bearing toes^27^, advanced ornithomimosaurs lack a hallux^28^, and some ornithomimosaurs have broad short ungulas^29^. Key to this study is the overall conservative morphology of most carnivorous theropod limbs, and limiting the considered taxa to carnivores, therefore, removes a substantial source of potential variation from the dataset. Finally, it has been argued that the limb proportions of herbivorous theropods have a different (nearly isometric) ontogeny^30^, and this difference would confound the later consideration of ontogenetic variation.

### Calculating cursorial-limb-proportion (CLP) score

To evaluate lower-leg proportions in the context of body mass requires a measureable osteological correlate of body mass. Femoral length is here used as that correlate. Femoral size has been found to be a reasonable indicator of body mass in multiple studies of both modern animals and dinosaurs^16^,^31^,^32^. Although other femoral dimensions (such as femur circumference and diameter) are slightly better size correlates than length^33^, length was selected as the measure of femur size, because femur length is seldom distorted by taphonomic factors and could be reliably measured from the largest number of specimens. By comparison, theropod femoral circumference and diameter are often impossible to reliably measure, because theropod femora are relatively thin walled and hollowed and are, therefore, prone to collapsing internally when fossilized and buried. As noted by Campione and Evans^33^, femur-length/mass scaling follows a roughly isometric pattern in modern animals, and multiple studies have shown femoral length to be among the least variable hindlimb dimensions^12^,^16^,^34^,^35^,^36^. Femoral length does, therefore, provide a generally reliable indicator of body mass, especially when compared among members of the same taxonomic group with the same general limb forms, and the morphology of non-avian theropod hindlimbs has long been recognised as highly conservative^20^. Additionally, it should be remembered, as pointed out by Carrano^37^ in his seminal work on dinosaur size evolution, that, because femoral length has an established linear relationship with body mass, femoral length serves as a valid proxy for body mass and allows the relative sizes of dinosaurs to be compared on the same scale (which is all that is needed in this study).

**Figure 2:**
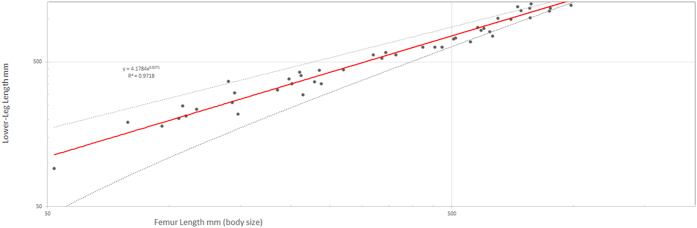
Log/log plot of femur vs. lower-leg length for the initial dataset of 53 theropod taxa. The red line denotes the best-fit power curve and the dotted lines denote the confidence interval.

The initial theropod dataset was used to create a bivariate plot^38^, with femur length and lower-leg length on either axis. A simple best-fit power curve was then applied to the plot (Fig. 2). All analyses were performed using Microsoft Excel 2013. This power curve (which is generated by Equation 1: *l* = 4.178*f*^ 0.8371^, where *l* is lower-leg length and *f* is femur length), is here interpreted as representing the normal relationship between body mass (approximated by femur length) and lower-leg length, as established by the dataset as a whole. As such, Equation 1 offers a way of predicting the lower-leg length of a particular species based on its femur length, and comparisons between a predicted lower-leg length and its true lower-leg length offers a way to quantitatively evaluate the relative abnormality of the lower-leg length of that species, irrespective of allometry. The percentage difference by which the true lower-leg length of a particular species differs from the lower-leg length predicted for that species by Equation 1 is here reported as the “cursorial-limb-proportion” (CLP) score of that species. This approach of deriving a comparative score of how a particular taxon differs from a prediction based on the absolute size of that taxon and analysis of a size-dependent relationship seen in a large sample of taxa is commonly used in assessments of allometricly influenced traits, with perhaps the most well-known example being the derivation of encephalization quotient (EQ) scores as a way of estimating animal intelligence from relative brain size^39^.

### Example

To better explain how the CLP scores were derived in this study, it may be helpful to briefly consider an example. The ceratosaurian theropod *Deltadromeus agilis* was given its name, which means “agile delta runner”, because Sereno *et al.*^40^ interpreted its elongate hindlimbs as being highly adapted for cursoriality. To evaluate the limb proportions of *D. agilis* using the methods of this study, the femur length (741 mm) is input into the equation for predicted lower-leg length (Equation 1) and yields a predicted length of 1055 mm. In actuality, *D. agilis* has a lower-leg length of 1134 mm. So, the true lower-leg length of *D. agilis* differs from its predicted lower-leg length by 79 mm. Thus the inference of Sereno *et al.*^40^ is here supported, as *D. agilis* is found to have a lower-leg that is 7.5% longer than would be “normal” for a theropod of its size (based on the relationship seen in the initial 53 taxa dataset) and is given a CLP score of +7.5. Note: if *D. agilis* had failed to live up to its name and had been found to have a lower-leg length that was abnormally short (below the predicted length), its CLP score would be reported as a negative value.

### Exploring consistency in multi-specimen taxa

Because calculating the CLP score for any particular species requires femoral, tibia, and metatarsal III length measurements, CLP scores can only be calculated from specimens with relatively complete hindlimbs. This limits the number of taxa that are able to contribute to the initial dataset. It also means that, for the vast majority of species, it is only possible to base the CLP score calculation on measurements taken from a single specimen. For the sake of consistency, all species in the initial dataset are represented only by single specimens (in instances where more than one potential specimen was available, the largest or the best preserved specimen was generally chosen). This imparts a potential source of error. In the first place, the initial dataset does not consider the amount of individual variation that may be present within a species. Secondly, and potentially more seriously, some species may be represented by specimens that are ontogenetically immature.

The few theropod species for which multiple specimens with sufficiently complete hindlimbs are known offer a chance to explore both the degree of individual variation in CLP score and the effect of ontogeny. Limb measurements were taken from multiple specimens of six theropod species (*Albertosaurus sarcophagus* n = 4, *Allosaurus fragilis* n = 8, *Coelophysis bauri* n = 10, *Gorgosaurus libratus* n = 6, *Herrerasaurus ischigualestensis* n = 7, and *Tyrannosaurus rex* n = 4) (Table 2).

## Results

**Figure 3:**
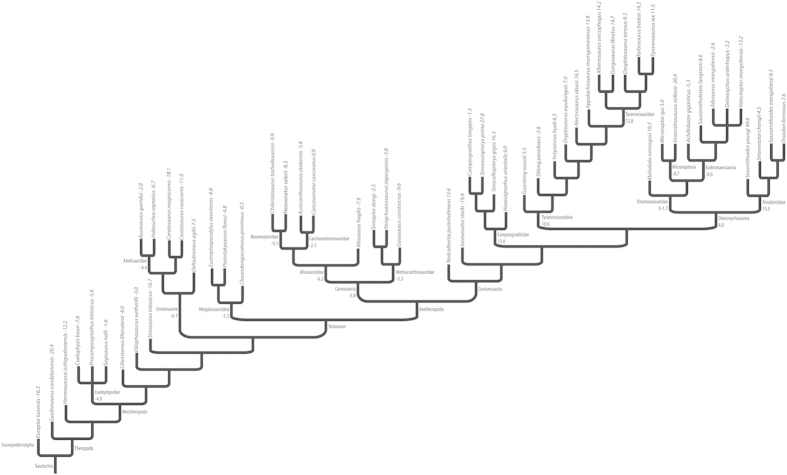
Theropod phylogeny, with CLP scores reported for individual species and average CLP scores reported for larger clades.

The CLP scores derived from the initial dataset are reported in Table 3, with all taxa arranged in rank order of CLP score, and in Fig. 3, with taxa arranged phylogenetically. The scores range from -20.4 to +40.6, with an average of +0.7.

**Figure 4:**
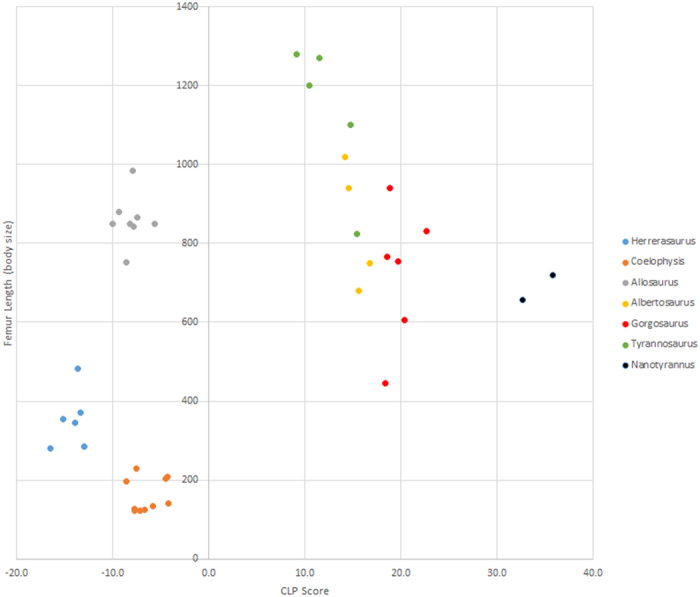
CLP scores vs. femur length for the multi-specimen and *Nanotyrannus* datasets. *Herrerasaurus ischigualestensis* n = 6, variance = 1.7, coefficient of variance = 0.09; *Coelophysis bauri* n = 10, variance = 2.6, coefficient of variance = −0.25; *Allosaurus fragilis* (n = 8, variance = 1.7, coefficient of variance = −0.16; *Albertosaurus sarcophagus* n = 4, variance = 1.4, coefficient of variance = 0.76; *Gorgosaurus libratus* n = 6, variance = 2.6, coefficient of variance = 0.08; *Tyrannosaurus rex* n = 5, variance = 7.5, coefficient of varianc = 0.22; *Nanotyrannus lancensis* n = 2, variance = 4.9, coefficient of variance = 0.06).

The CLP scores derived from the multi-specimen dataset are presented in Table 2 and Fig. 4. The highest variation in the scores within any species was found in the four specimens of *Tyrannosaurus rex*—scores ranged from +15.5 to +9.1, a difference of 6.4. However, a series of F-tests confirm that the amount of variance seen in the *Tyrannosaurus rex* data is not significantly greater than that observed in any of the other multi-specimen taxa. The greatest deviation of any score from the mean score of its species was found in the *Tyrannosaurus rex* specimen MOR 555, which deviates from the mean by 3.2. This suggests that, when interpreting the scores derived from the initial dataset, it is reasonable to assume that the reported CLP scores may deviate from the average CLP score of that species by as much +/−3.2.

Of the eight oldest and phylogenetically least-derived theropods included in the initial dataset, none had a positive CLP score. *Guaibasaurus candelariensis*, and *Herrerasaurus ischigualestensis*, the most basal dinosaurs included in the study, both have scores below −10 (implicating them as among the least cursorially adapted). These results contradict previous interpretations that some early theropods are examples of highly cursorial forms, and suggests that such interpretations were misled by the effect of allometry and the relative small size of these early theropods. Instead, the primitive theropod condition appears to have been hindlimb proportions that are relatively non-cursorial. Evidence of high cursorial limb proportions were found among deinonychosaurs, tyrannosauroids, compsognathids, and also found in the non-coelurosaurian theropods *Concavenator corcovatus* and *Deltadromeus agilis*.

Note that we have treated *Guaibasaurus candelariensis*, and *Herrerasaurus ischigualestensis* as theropods. There is currently debate over whether these taxa belong within the Theropoda proper or if they fall outside it^41^,^42^,^43^,^44^. Additionally, it has been argued that *Guaibasaurus candelariensis* may have closer affinities to the sauropodamorpha^45^,^46^. Although *Guaibasaurus candelariensis* has the lowest CLP score in the dataset, it is not a statistical outlier (according to a Grubbs’ test).

## Discussion and Additional Analyses

### Tyrannosauroids

One clear result from the initial dataset CLP score calculations is that tyrannosauroids have high CLP scores. Among the sampled tyrannosauroids, the basal taxa *Dilong paradoxus*, *Dryptosaurus aquilunguis*, *Guanlong wucaii*, and *Yutyrannus huali* have the lowest scores, while the more advanced tyrannosauroids *Alectrosaurus olsoni* and *Appalachiosaurus montgomeriensis* and all tyrannosaurs have much higher scores. This confirms previous assessments that tyrannosauroids are characterised by proportionately elongate hindlimbs and that lower-leg length became more exaggerated in later and more advanced forms^11^,^12^,^20^. The development of high CLP scores in derived tyrannosauroids is consistent with the evolution of an arctometatarsus. The arctometatarsus is a modified metatarsal form that has been linked to fast linear locomotion^12^,^47^ and enhanced agility^48^,^49^,^50^.

That tyrannosauroids have exceptionally elongate lower-legs is a factor that may modify how the results of this study should be interpreted. Because of their more recent heritage and the resulting high abundance of more complete specimens, tyrannosauroids make a large contribution to the initial dataset (eleven taxa, accounting for more than 20%). In particular, tyrannosauroids are disproportionately represented among the extremely large theropods in the initial dataset (tyrannosauroids account for seven of the thirteen theropods with femur length greater than 750 mm and four of the six theropods with femur length greater than 1000 mm). This high concentration of proportionately long legged but extremely large theropods may have skewed the dataset and had undue influence on the derivation of the predicted lower-leg length equation. To test this possibility, the tyrannosauroids data was separated from the initial dataset and the resulting two new datasets were subjective to an analysis of covariance (ANCOVA) using R statistical software. The result suggests that the tyrannosauroid data does have a significant influence (F =  21.06, p > 0.001). This indicates that special caution is warranted when interpreting the scores of species found by this study to have negative or “abnormally” low CLP scores. In fact, the proportions of these species may actually be closer to the norm or even above it, but have received a negative score because they are being considered within a dataset that has a high concentration of the extremely leggy tyrannosauroids. In particular, the low scores reported for other large theropods should be interpreted cautiously, and the method and approach here outlined will benefit from the future addition of more large non-tyrannosauroid taxa, but, at present, sufficiently complete specimens from such taxa are lacking.

### Deinonychosaurs

Gatesy and coauthors^18^,^51^,^52^,^53^,^54^ observed that a major change in hindlimb locomotive style occurred during the evolution of birds and their close relatives: the size and importance of the caudofemoral musculature was greatly reduced and the importance of knee flexion increased, while the importance of femoral retraction decreased. Although it was originally hypothesised that this change in locomotive musculature and emphasis occurred gradually across the whole of the theropod lineage, caudofemoral musculature remained important and unreduced in many coelurosaurian groups^55^,^56^. However, even the most basal deinonychosaurs show evidence of substantial caudofemoral reduction and are inferred to have begun the corresponding change in locomotor style^57^. This means that the CLP scores calculated for deinonychosaurs should be interpreted with special caution. As seen in modern birds, greater emphasis on knee flexion requires, if stride length and speed are not to be diminished, greater elongation of the lower leg and concomitantly reduced femora^53^,^58^.

Because increased emphasis on knee flexion generally requires proportionately longer metatarsals and shorter femora to still accomplish high speed running and because deinonychosaurs are classically regarded as among the more cursorily adapted theropods, deinonychosaurs would be doubly expected to have high CLP scores. However, this expectation is only partially met. All four troodontid species were found to have high CLP scores (ranging from +4.5 to +40.6 – the highest score of any of the considered taxa), and a Grubbs’ test found *Sinornithoides youngi*, which has the highest CLP score in the dataset (40.6) to be a significant outlier (Z = 3.337, critical Z =  3.151). This suggests that at least some troodontids had adapted avian-like limb proportions. However, the CLP scores of the eight dromaeosaurs were decidedly mixed (ranging from −20.4 to +18.5). Of these, *Mahakala omnogovi*, *Microraptor gui*, and *Saurornitholestes langstoni* have extremely high scores, while the CLP scores of the other five dromaeosaurs are all negative.

That a majority of the considered dromaeosaurs were found to have low scores indicates dromaeosaurs, as a group, did not undergo strong adaptive limb specialization for high-speed running. Indeed, given the reduced caudofemoral musculature of dromaeosaurs and the higher CLP scores of most compsognathids and tyrannosauroids (both more basal coelurosaurian groups), the opposite seems true: a majority of dromaeosaurs appear to have undergone a de-emphasis on cursorial limb proportions and to have been exposed to strong selective pressures favoring reduced running ability. These results are largely consistent with those of Carrano (1990)^11^. As in tyrannosauroids, the deinonychosaurs CLP scores are consistent with the presence/absence of an arctometatarsus: the consistently high scoring troodontids possess an arctometatarsus, while the dromaeosaurs do not.

### Ontogenetic variation and controversial taxa

Among the multi-specimen dataset were individuals of the same species that differed from each other substantially in terms of femur length, and, therefore, assumed body mass. Although absolute size is not always an indicator of relative age, it is reasonable to assume that many of the smaller specimens probably represent younger individuals. For instance, the largest of the *Coelophysis bauri* specimens (CMNH 10971a) has a femur that is more than 53% longer than the smallest (AMNH 7246), and the largest of the *Herrerasaurus ischigualestensis* specimens (PVL 2566) has a femur that is more than 58% longer than the smallest (MACN 18.060). Four of the tyrannosaur specimens are known juveniles (the *Albertosaurus sarcophagus* specimen NMC 11315, the *Gorgosaurus libratus* specimens AMNH 5423 and FMNH PR 2211, and the *Tyrannosaurus rex* specimen LACM 23845). Of these, FMNH PR 2211 has a femur length that is less than half that of NMC 11593 (implying an order of magnitude difference in likely bodyweight). Yet, the CLP scores calculated for even these exceptionally large and small specimens do not strongly vary from each other and fall within or near the range of scores calculated from other more moderately sized members of the same species (Fig. 4). The CLP score of a particular theropod species, therefore, appears to neither increase nor decrease with mass and age. This indicates that the widely documented changes in theropod hindlimb proportions over ontogeny can be largely explained by factors simply relating to growth in body mass.

Aside from indicating a generalized growth pattern across theropods, the recognition that, with respect simply to body mass, theropod hindlimb proportions follow roughly the same trend ontogenetically as interspecifically has several implications. First, it suggests that even if some specimens included in the initial dataset are immature, the CLP scores derived for those specimens are not likely to be misrepresentative. Second, it means that comparing CLP scores offers a potential independent method for assessing the validity of novel taxa erected based on immature or age-indeterminate specimens, which are suspected of belonging to pre-existing species, particularly when differences in limb proportions are hypothesised to be discriminating characters.

For example, the controversial Late Cretaceous tyrannosaur *Nanotyrannus lancensis* has been interpreted by some as a separate genus^59^,^60^,^61^,^62^ and by others as a junior synonym of *Tyrannosaurus rex*^16^,^63^,^64^. Arguments that favor the synonymy of *N. lancensis* and *T. rex* center on the interpretation of the various traits that appear to differ between *N. lancensis* and *T. rex* (which include various proportions of the skull, a small foramen in the quadratojugal, braincase morphology, tooth counts, and the form of the glenoid) as being ontogenetically dependent and indicating that all alleged *N. lancensis* specimens are, in actuality, immature specimens of *T. rex*. There has been much discussion over the legitimacy of *N. lancensis* within the literature, but there will be no attempt here to summarize the points and counterpoints of, and to, the various arguments made by both camps (instead, readers are directed to consult Carr^63^ and Larson^59^). These arguments include a variety of anatomical proportions and characters, with Carr^64^ emphasising synapomorphies and *N. lancensis* diagnoses generally focusing on differences between it and *T. rex*. The elongate hindlimb proportions of specimens referred to *N. lancensis* have been specifically implicated in the debate as a trait that distinguishes *N. lancensis* and, alternatively, as a trait that can be explained away as simply reflecting immaturity.

The allegedly elongate hindlimbs of *Nanotyrannus lancensis* also have potential paleoecological implications. Excluding *N. lancensis*, *Tyrannosaurus rex* is the only large carnivorous theropod known from the uppermost Maastrichtian beds of North America. This implies a lower diversity in large predators than is seen in most other well sampled dinosaur faunas. Furthermore, it has been postulated that the more elongate hindlimbs of *N. lancensis* reflect a form of predatory ecological niche partitioning between *N. lancensis* and *T. rex*. Bakker^65^ has suggested that the two tyrannosaurs are analogous to modern lions and cheetahs, with the smaller, more gracile, and longer legged *N. lancensis* being adapted for high-speed running. Such a comparison implies that *N. lancensis* should have proportionately more elongate hindlimbs and should, therefore, be predicted to have a much higher CLP score than *T. rex*.

To test this prediction, leg measurements were taken from two specimens that have been referred to *Nanotyrannus lancensis.* The first of these specimens is BMRP 2002.4.1 (“Jane”). The second is BHI-6437, a 3-D digital specimen produced through photogrammetry^60^ and accessioned in the digital collections of the Black Hills Institute of Geological Research (see acknowledgments). The measurements and resulting CLP scores for BMRP 2002.4.1 and BHI-6437 (Table 4) are very close to one another (35.8 and 32.7, respectively), and the scores of both these specimens fall well above the range of scores established from the four specimens of *Tyrannosaurus rex*. Moreover, the scores of BMRP 2002.4.1 and BHI-6437 exceed the range of scores established by any of the other tyrannosauroids, including similarly sized and immature *Albertosaurus* and *Gorgosaurus* specimens (Fig. 4). The prediction that the alleged *Nanotyrannus lancensis* specimens should show limb proportions indicative of high cursorial adaptation is, therefore, met. Indeed, the two *N. lancensis* scores exceed those of any other non-avialan theropod included in the initial dataset, making *N. lancensis* arguably the most cursorily adapted of all non-avialan carnivorous theropods.

Nevertheless, some caution is warranted in the interpretation of the exceptionally high CLP scores here reported for the two alleged *Nanotyrannus lancensis* specimens. Although the score of BMRP 2002.4.1 and BHI-6437 exceed those of adult *Tyrannosaurus rex* specimens and those of juvenile specimens of other tyrannosaurs, there is, as yet, no clearly identified juvenile *T. rex* specimen with limb proportions different from the referred *N. lancensis* specimens. It could, therefore, still be argued that *T. rex* and *N. lancensis* are synonymous, and that these results simply show that juvenile *T. rex* possessed abnormally cursorial limb proportions that became altered over ontogeny. This is an interpretation with its own significant ecological implications – perhaps for ontogenetic diet shifts and adult vs. juvenile niche partitioning. However, such an interpretation is an argument for a special case, because, in at least two other genera of large-bodied tyrannosaurs (*Albertosaurus* and *Gorgosaurus*), it is known that no similar changes in limb proportions occur. Establishing growth series for other large tyrannosaurs, including the closely related *Tarbosaurus*, will help further address this challenge.

### Conclusion

Accounting for the influence of allometry permits cursorial hindlimb proportions to be scored across all carnivorous theropods, regardless of body mass. CLP scores are generally low among early primitive theropods but are high in more derived forms, including both the small-bodied compsognathids and the large-bodied tyrannosaurs. This supports previous arguments that coelurosaurs are characterised by highly cursorial limb proportions^11^,^12^ and supports more general inferences of increased relative limb elongation throughout the evolutionary history of predatory theropods^66^. However, dromaeosaurs constitute an exception, as several dromaeosaur taxa appear to have strongly reduced cursorial limb proportions.

That the same allometric correcting method derived from interspecific comparisons also appears effective in intraspecific ontogenetic comparisons, indicates that much of the ontogenetic limb variation previously reported within different theropod taxa can be explained in terms of simple allometry. Although in the tyrannosaurs *Albertosaurus* and *Gorgosaurus*, small bodied juveniles were found to fall within the same CLP-score range as large-bodied adults, CLP scores calculated for the tyrannosaur *Nanotyrannus* fell well outside the range of scores calculated for *Tyrannosaurus*. This result suggests that the proportionately elongate lower legs of *Nanotyrannus* are not allometrically equivalent to those of *Tyrannosaurus* and are, therefore, a legitimate character to cite as a morphological discriminator between the two. This illustrates how CLP scores may in future studies be used in taxonomic assessments of juvenile specimens.

Unlike many previous attempts at estimating maximum running speeds in theropods and other approaches to allometric assessments of cursorial adaptations, the method here outlined is simple and Equation 1 can easily be applied to other theropod taxa. This method offers a way to quantify the degree of hindlimb elongation in descriptions of new theropod taxa, such that cursoriality can be quantitatively and more accurately assessed. Hopefully this method will be refined and utilized as new specimens become available, and the methodology will be applied to other dinosaur groups.

## Additional Information

**How to cite this article**: Persons IV, W. S. and Currie, P. J. An approach to scoring cursorial limb proportions in carnivorous dinosaurs and an attempt to account for allometry. *Sci. Rep.* **6**, 19828; doi: 10.1038/srep19828 (2016).

## Figures and Tables

**Figure 1:**
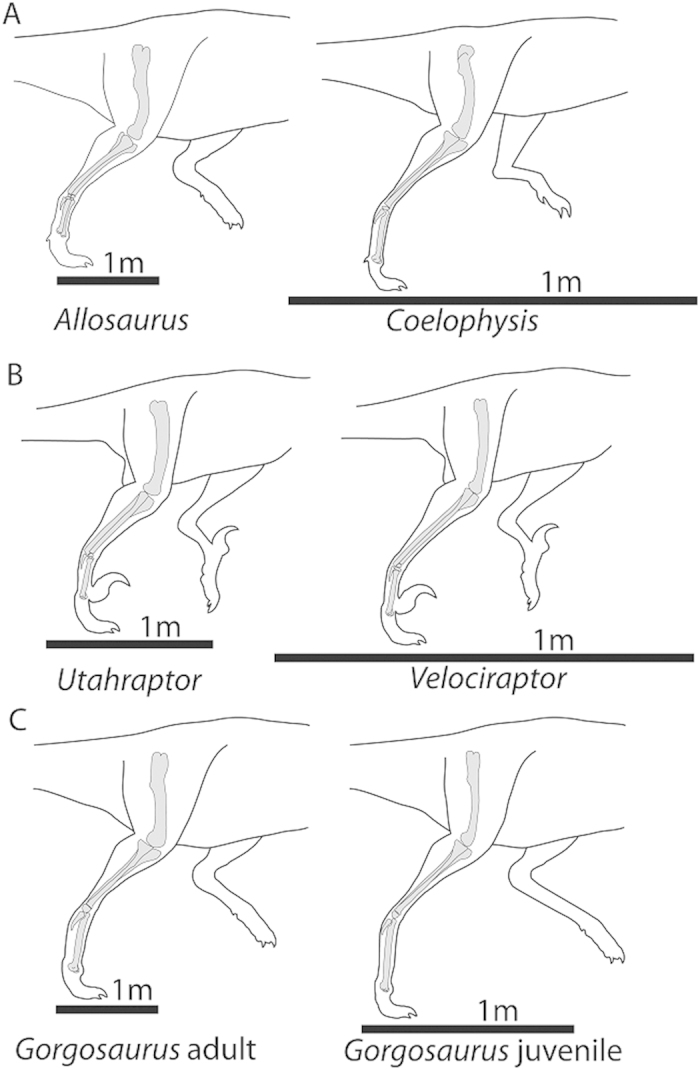
The general observation that smaller-bodied non-avian theropods tend to have proportionately longer lower legs holds true across comparisons between distantly related taxa (A), closely related taxa (B), and ontogenetic stages within a single taxon (C). All illustrations scaled to the same proximodistal femur length.

**Table 1:** Hindlimb measurements form the initial theropod dataset (all measurements in mm).

Species	ID	Femur Length	Tibia Length	Metatarsal III Length	Lower-leg length
Basal Theropods					
* Coelophysis bauri*	CMNH 10971a	229	227	138	365
* Dilophosaurus wetherilli*	UCMP 77270	600	560	298	858
* Guaibasaurus candelariensis*	MCN-PV 2355	214	202	95	297
* Herrerasaurus ischigualestensis*	PVL 2566	473	413	223	636
* Liliensternus liliensterni*	HMN R1291	424	415	220	635
* Procompsognathus triassicus*	SMNS 12951	96	110	70	180
* Segisaurus halli*	UCMP 32101	143	164	98	262
* Sinosaurus triassicus*	KMV 8701	556	460	231	691
Ceratosaurs					
* Aucasaurus garridoi*	MCF-PBPH-236	700	640	346	986
* Ceratosaurus magnicornis*	MWC 1.1	630	520	234	754
* Ceratosaurus nasicornis*	USNM 4735	620	555	254	809
* Deltadromeus agilis*	SGM-Din 2	741	700	434	1134
* Indosuchus raptorius*	ISI R 401-454	872	795	333	1128
Megalosauroids					
* Chuandongocoelurus primitivus*	CCG 20010	201	231	122	353
* Eustreptospondylus oxoniensis*	OUM J13558	510	495	240	735
* Piatnitzkysaurus floresi*	PVL 4073	590	541	289	830
Carnosaurs					
* Acrocanthosaurus atokensis*	NCSM 14345	1120	952	453	1405
* Allosaurus fragilis*	AMNH 290	985	810	423	1233
* Chilantaisaurus tashuikouensis*	IVPP V.2884	1190	954	460	1414
* Concavenator corcovatus*	MCCM-LH 6666	580	580	287	867
* Gasosaurus constructus*	IVPP V.7264	454	382	251	633
* Neovenator salerii*	MIWG 6348/BMNH R.10001	780	670	340	1010
* Sinraptor dongi*	IVPP 87002	875	772	410	1182
* Yangchuanosaurus zigongensis*	IVPP V.239	364	360	200	560
Basal Coelurosaurs					
* Juravenator starki*	JME Sch 200	52	58	34	92
* Nedcolbertia justinhofmanni*	TMP 96.90.2 (cast of CEUM 5071)	145	199	107	306
Compsognathids					
* Compsognathus longipes*	MNHN CNJ 79	110	130	81	211
* Huaxiagnathus orientalis*	NIGP Mv97	235	280	160	440
* Sinocalliopteryx gigas*	JMP-V-05-8-01	210	280	147	427
* Sinosauropteryx prima*	GMV 2124	108	152	96	248
Tyrannosauroids					
* Albertosaurus sarcophagus*	ROM 807	1020	980	595	1575
* Alectrosaurus olsoni*	AMNH 6554	727	730	480	1210
* Appalachiosaurus montgomeriensis*	RMM6670	786	781	482	1263
* Daspletosaurus torosus*	MOR 590	865	815	498	1313
* Dilong paradoxus*	IVPP V14243	185	203	118	321
* Dryptosaurus aquilunguis*	TMP 84.181.2 (cast of ANSP 9995/10006)	778	796	380	1176
* Gorgosaurus libratus*	NMC 2120	1030	980	615	1595
* Guanlong wucaii*	IVPP V14531	343	395	189	584
* Tarbosaurus baatar*	MPC-D100/63	1020	980	595	1575
* Tyrannosaurus rex*	CM 9380 (cast of AMNH 973)	1269	1166	680	1846
* Yutyrannus huali*	ZCDM V5001, IVPP FV1961	650	655	350	1005
Dromaeosaurs					
* Achillobator giganticus*	MNUFR-15	505	490	234	725
* Adasaurus mongoliensis*	MPC-D100/20	270	295	147	442
* Deinonychus antirrhopus*	MCZ 4371	336	368	164	532
* Mahakala omnogovi*	MPC 100/1033	79	110	82	192
* Microraptor gui*	QM V1002	108	145	72	217
* Saurornitholestes langstoni*	TMP 88.121.39	212	285	117	402
* Sinornithosaurus millenii*	IVPP V.12811	148	125	93	218
* Velociraptor mongoliensis*	IGM 100/986	238	255	99	354
Troodontids					
* Saurornithoides mongoliensis*	AMNH 6516	198	243	139	382
* Sinornithoides youngi*	IVPP V.9612	140	191	177	368
* Sinovenator changii*	IVPP V12615	117	149	86	235
* Troodon formosus*	MOR 748	320	352	210	562

**Table 2:** Hindlimb measurements form the multi-specimen dataset (all measurements in mm).

ID	Femur Length	Tibia Length	Metatarsal III Length	Lower-leg length
*Herrerasaurus ischigualestensis*				
MACN 18.060	280.6	259	132	391
MACN 18.090	286	280	134	414
PVL 2054	370	335	176	511
PVL 2566	482	415	221	636
PVSJ 373	345	315	164	479
PVSJ 373	354	318	165	483
*Coelophysis bauri*				
AMNH 7223	209	224	126	350
AMNH 7224	203	221	120	341
AMNH 7229	135	154	85	239
AMNH 7232	141	157	95	252
AMNH 7233	126	140	81	221
AMNH 7246	122	136	79	215
AMNH 7247	125	138	84	222
AMNH 7249	196	207	110	317
CMNH 10971a	229	227	138	365
MNA V3318	123	136	82	218
*Allosaurus fragilis*				
AMNH 290	985	810	423	1233
AMNH 324	850	738	327	1065
AMNH 6125	850	732	355	1087
CM 11844	843	724	360	1084
USNM 4734	753	658	320	978
UUVP 6000	865	738	374	1112
UUVP 60001	850	745	372	1117
UUVP 6000r	880	730	375	1105
*Albertosaurus sarcophagus*				
NMC 11315	680	690	445	1135
ROM 807	1020	980	595	1575
TMP 1981.10.1	940	900	575	1475
TMP 1985.98.1	750	770	475	1245
*Gorgosaurus libratus*				
AMNH 5423	605	640	432	1072
TCMI 2001.89.1	830	885	538	1423
FMNH PR 2211	445	472	343	815
NMC 11593	940	925	605	1530
ROM 1247	765	785	500	1285
TMP 91.163.001	755	770	513	1283
*Tyrannosaurus rex*				
BHI 6230	1100	1025	660	1685
CM 9380 (cast of AMNH 973)	1269	1166	680	1846
MOR 555	1280	1150	670	1820
RTMP 81.12.1 (cast of NMC 9950)	1200	1095	650	1745
LACM 23845	825	825	508	1333

**Table 3:** Cursorial-limb-proportion (CLP) scores from the initial dataset.

*Sinornithosaurus millenii*	−20.4	*Segisaurus halli*	−1.6
*Guaibasaurus candelariensis*	−20.4	*Compsognathus longipes*	−1.3
*Juravenator starki*	−19.4	*Chuandongocoelurus primitivus*	−0.3
*Ceratosaurus magnicornis*	−18.1	*Concavenator corcovatus*	0.9
*Sinosaurus triassicus*	−16.7	*Microraptor gui*	3.0
*Velociraptor mongoliensis*	−13.2	*Sinovenator changii*	4.5
*Herrerasaurus ischigualestensis*	−12.2	*Guanlong wucaii*	5.5
*Ceratosaurus nasicornis*	−11.0	*Yutyrannus huali*	6.3
*Chilantaisaurus tashuikouensis*	−9.9	*Dryptosaurus aquilunguis*	7.0
*Gasosaurus constructus*	−9.6	*Deltadromeus agilis*	7.5
*Neovenator salerii*	−8.3	*Troodon formosus*	7.6
*Allosaurus fragilis*	−7.9	*Saurornitholestes langstoni*	8.6
*Coelophysis bauri*	−7.6	*Huaxiagnathus orientalis*	9.0
*Indosuchus raptorius*	−6.7	*Saurornithoides mongoliensis*	9.3
*Acrocanthosaurus atokensis*	−5.8	*Daspletosaurus torosus*	9.3
*Procompsognathus triassicus*	−5.6	*Tyrannosaurus rex*	11.5
*Achillobator giganticus*	−5.3	*Nedcolbertia justinhofmanni*	13.6
*Piatnitzkysaurus floresi*	−4.8	*Appalachiosaurus montgomeriensis*	13.9
*Eustreptospondylus oxoniensis*	−4.8	*Albertosaurus sarcophagus*	14.2
*Liliensternus liliensterni*	−4.0	*Tarbosaurus baatar*	14.2
*Yangchuanosaurus zigongensis*	−3.8	*Gorgosaurus libratus*	14.7
*Dilophosaurus wetherilli*	−3.0	*Sinocalliopteryx gigas*	16.3
*Dilong paradoxus*	−2.8	*Alectrosaurus olsoni*	16.5
*Adasaurus mongoliensis*	−2.6	*Sinosauropteryx prima*	17.8
*Sinraptor dongi*	−2.5	*Mahakala omnogovi*	18.5
*Deinonychus antirrhopus*	−2.2	*Sinornithoides youngi*	40.6
*Aucasaurus garridoi*	−2.0		

**Table 4:** Limb measurements and CLP scores from the *Nanotyrannus lancensis* dataset (all measurements in mm).

ID	Femur Length	Tibia Length	Metatarsal III Length	Lower-leg length	Leg Score
BMRP 2002.4.1	720	836	563	1399	35.8
BHI-6437	657	720	546	1266	32.7

## References

[b1] Alexander R. Estimates of speeds of dinosaurs. Nature 261, 129–130 (1976).

[b2] Thulborn R. A. Preferred gaits of bipedal dinosaurs. Alcheringa 8, 243–252 (1984).

[b3] Bates K. T., Manning P. L., Margetts L. & Sellers W. I. Sensitivity analysis in evolutionary robotic simulations of bipedal dinosaur running. Journal of Vertebrate Paleontology 30, 458–466 (2010).

[b4] Sellers W. I. & Manning P. L. Estimating dinosaur maximum running speeds using evolutionary robotics. Proceedings of the Royal Society of London B: Biological Sciences 274, 2711–2716 (2007). 10.1098/rspb.2007.0846 PMC2279215 17711833

[b5] Hutchinson J. R. & Gatesy S. M. Dinosaur locomotion: beyond the bones. Nature 440, 292–294 (2006). 16541062 10.1038/440292a

[b6] Alexander R. Optimization and gaits in the locomotion of vertebrates. Physiol. Rev 69, 29–64 (1989). 10.1152/physrev.1989.69.4.1199 2678167

[b7] Heglund N. C. & Cavagna G. A. Efficiency of vertebrate locomotory muscles. Journal of Experimental Biology 115, 283–292 (1985). 4031770 10.1242/jeb.115.1.283

[b8] Pennycuick C. On the running of the gnu (Connochaetes taurinus) and other animals. The Journal of Experimental Biology 63, 775–799 (1975).

[b9] Biewener A. A. Allometry of quadrupedal locomotion: the scaling of duty factor, bone curvature and limb orientation to body size. Journal of Experimental Biology 105, 147–171 (1983). 6619724 10.1242/jeb.105.1.147

[b10] Christiansen P. Locomotion in terrestrial mammals: the influence of body mass, limb length and bone proportions on speed. Zoological Journal of the Linnean Society 136, 685–714 (2002).

[b11] Carrano M. What, if anything, is a cursor? Categories versus continua for determining locomotor habit in mammals and dinosaurs. Journal of Zoology 247, 29–42 (1999).

[b12] Holtz T. R. Jr The arctometatarsalian pes, an unusual structure of the metatarsus of Cretaceous Theropoda (Dinosauria: Saurischia). Journal of Vertebrate Paleontology 14, 480–519 (1995).

[b13] Scott K. M. Allometric trends and locomotor adaptations in the Bovidae. Bulletin of the AMNH, 179, 197–288 (1985).

[b14] Bakker R. T. The dinosaur heresies (Penguin, 1988).

[b15] Bakker R. T. The deer flees, the wolf pursues: incongruities in predator-prey coevolution In Coevolution (eds. Futuyma D. J., Slatkin M.) 35–382 (Sunderland, 1983).

[b16] Currie P. J. Allometric growth in tyrannosaurids (Dinosauria: Theropoda) from the Upper Cretaceous of North America and Asia. Canadian Journal of Earth Sciences 40, 651–665 (2003).

[b17] Garland T. & Janis C. M. Does metatarsal/femur ratio predict maximal running speed in cursorial mammals? Journal of Zoology 229, 133–151 (1993).

[b18] Gatesy S. M. & Middleton K. M. Bipedalism, flight, and the evolution of theropod locomotor diversity. Journal of Vertebrate Paleontology 17, 308–329 (1997).

[b19] Carrano M. T. & Sidor C. A. Theropod hind limb disparity revisited: Comments on Gatesy and Middleton (1997). Journal of Vertebrate Paleontology 19, 602–605 (1999).

[b20] Farlow J. O., Gatesy S. M., Holtz T. R., Hutchinson J. R. & Robinson J. M. Theropod locomotion. American Zoologist 40, 640–663 (2000).

[b21] Sellers W. I. & Manning P. L. Estimating dinosaur maximum running speeds using evolutionary robotics. Proceedings of the Royal Society B: Biological Sciences 274, 2711–2716 (2007). 17711833 10.1098/rspb.2007.0846 PMC2279215

[b22] Christiansen P. Strength indicator values of theropod long bones, with comments on limb proportions and cursorial potential. Gaia 15, 241–255 (1998).

[b23] Farlow J. O., Smith M. B. & Robinson J. M. Body mass, bone “strength indicator”, and cursorial potential of Tyrannosaurus rex. Journal of Vertebrate Paleontology 15, 713–725 (1995).

[b24] Hutchinson J. R. & Garcia M. *Tyrannosaurus* was not a fast runner. Nature 415, 1018–1021 (2002). 11875567 10.1038/4151018a

[b25] Carrano M. T. Implications of limb bone scaling, curvature and eccentricity in mammals and non‐avian dinosaurs. Journal of Zoology 254, 41–55 (2001).

[b26] Coombs W. P. Jr Theoretical aspects of cursorial adaptations in dinosaurs. Quarterly Review of Biology, 393–418 (1978).

[b27] Fiorillo A. R. & Adams T. L. A therizinosaur track from the Lower Cantwell Formation (Upper Cretaceous) of Denali National Park, Alaska. Palaios 27, 395–400 (2012).

[b28] Barsbold R. & Osmólska H. Ornithomimosauria In The Dinosauria (eds. Dodson P. & Osmólska H.) 225–244 (University of California Press, 1990).

[b29] Lee Y.-N. *et al.* Resolving the long-standing enigmas of a giant ornithomimosaur *Deinocheirus* mirificus. Nature 515, 257–360 (2014). 25337880 10.1038/nature13874

[b30] Lü J. *et al.* Chicken-sized oviraptorid dinosaurs from central China and their ontogenetic implications. Naturwissenschaften 100, 165–175 (2013). 23314810 10.1007/s00114-012-1007-0

[b31] Christiansen P. Long bone scaling and limb posture in non-avian theropods: evidence for differential allometry. Journal of Vertebrate Paleontology 19, 666–680 (1999).

[b32] Farlow J. O., Hurlburt G. R., Elsey R. M., Britton A. R. & Langston W. Jr Femoral dimensions and body size of Alligator mississippiensis: estimating the size of extinct mesoeucrocodylians. Journal of Vertebrate Paleontology 25, 354–369 (2005).

[b33] Campione N. E. & Evans D. C. A universal scaling relationship between body mass and proximal limb bone dimensions in quadrupedal terrestrial tetrapods. Bmc Biology 10, 60 (2012). 22781121 10.1186/1741-7007-10-60 PMC3403949

[b34] Russell D. A. Tyrannosaurs from the Late Cretaceous of western Canada (Queen’s Printer, 1970).

[b35] Currie P. J. & Zhao X.-J. A new carnosaur (Dinosauria, Theropoda) from the Jurassic of Xinjiang, People’s Republic of China. Canadian Journal of Earth Sciences 30, 2037–2081 (1993).

[b36] Rosenberg D. & Dodson P. An allometric analysis of dinosaur skeletons. Journal of Vertebrate Paleontology 16, 61 (1996).

[b37] Carrano M. T. Body-size evolution in the dinosauria In Amniote paleobiology: perspectives on the evolution of mammals, birds, and reptiles (eds Carrano M. T. *et al.*) Ch. 8, 225–268 (University of Chicago Press, 2006).

[b38] Sokal R. R. & Rohlf F. J. Biometry, The Principles and Practice of Statistics in Biological Research (WH Freeman & Co. 1969)

[b39] Jerison H. J. The theory of encephalization. Annals of the New York Academy of Sciences 299, 146–160 (1977). 280197 10.1111/j.1749-6632.1977.tb41903.x

[b40] Sereno P. C. *et al.* Predatory dinosaurs from the Sahara and Late Cretaceous faunal differentiation. Science 272, 986–991 (1996). 8662584 10.1126/science.272.5264.986

[b41] Sereno P. C. & Novas F. E. The skull and neck of the basal theropod Herrerasaurus ischigualastensis. Journal of Vertebrate Paleontology 13, 451–476 (1994).

[b42] Sereno P. C. & Novas F. E. The complete skull and skeleton of an early dinosaur. Science 258, 1137–1137 (1992). 17789086 10.1126/science.258.5085.1137

[b43] Novas F. E. New information on the systematics and postcranial skeleton of Herrerasaurus ischigualastensis (Theropoda: Herrerasauridae) from the Ischigualasto Formation (Upper Triassic) of Argentina. Journal of Vertebrate Paleontology 13, 400–423 (1994).

[b44] Sereno P. C., Forster C. A., Rogers R. R. & Monetta A. M. Primitive dinosaur skeleton from Argentina and the early evolution of Dinosauria. Nature 361, 64–66 (1993).

[b45] Sereno P. C., Martínez R. N. & Alcober O. A. Osteology of Eoraptor lunensis (Dinosauria, Sauropodomorpha). Journal of Vertebrate Paleontology 32, 83–179 (2012).

[b46] Ezcurra M. D. A new early dinosaur (Saurischia: Sauropodomorpha) from the Late Triassic of Argentina: a reassessment of dinosaur origin and phylogeny. Journal of Systematic Palaeontology 8, 371–425 (2010).

[b47] Wilson M. C. & Currie P. J. Stenonychosaurus inequalis (Saurischia: Theropoda) from the Judith River (Oldman) Formation of Alberta: new findings on metatarsal structure. Canadian Journal of Earth Sciences 22, 1813–1817 (1985).

[b48] Snively E. Functional morphology of the tyrannosaurid arctometatarsus (University of Calgary, 2000).

[b49] Snively E. & Russell A. P. Kinematic model of tyrannosaurid (Dinosauria: Theropoda) arctometatarsus function. Journal of Morphology 255, 215–227 (2003). 12474267 10.1002/jmor.10059

[b50] Snively E., Russell A. P. & Powell G. L. Evolutionary morphology of the coelurosaurian arctometatarsus: descriptive, morphometric and phylogenetic approaches. Zoological Journal of the Linnean Society 142, 525–553 (2004).

[b51] Gatesy S. M. Caudefemoral musculature and the evolution of theropod locomotion. Paleobiology 16, 170–186 (1990).

[b52] Gatesy S. M. & Dial K. P. Locomotor modules and the evolution of avian flight. Evolution 50, 331–340 (1996). 10.1111/j.1558-5646.1996.tb04496.x 28568886

[b53] Gatesy S. M. Functional evolution of the hind limb and tail from basal theropods to birds In Functional morphology in vertebrate paleontology (ed. Thomason J.), 219–234 (Cambridge University Press, 1995).

[b54] Hutchinson J. R. & Gatesy S. M. Adductors, abductors, and the evolution of archosaur locomotion. Paleobiology 26, 734–751 (2009).

[b55] Persons W. S. IV & Currie P. J. The tail of *Tyrannosaurus*: reassessing the size and locomotive importance of the M. caudofemoralis in non‐avian theropods. The Anatomical Record 294, 119–131 (2011). 21157923 10.1002/ar.21290

[b56] Persons W. S. IV, Currie P. J. & Norell M. A. Oviraptorosaur tail forms and functions. Acta Palaeontologica Polonica 59, 553–567 (2013).

[b57] Persons W. S. & Currie P. J. Dragon tails: convergent caudal morphology in winged archosaurs. Acta Geologica Sinica 86, 1402–1412 (2012).

[b58] Christiansen P. & Bonde N. Limb proportions and avian terrestrial locomotion. Journal für Ornithologie 143, 356–371 (2002).

[b59] Larson P. The case for *Nanotyrannus* In Tyrannosaurid paleobiology (eds Parrish J. *et al.*) 15–53 (Indiana University Press, 2013).

[b60] Larson P. The validity of *Nanotyrannus lancensis* (Theropoda, Lancian - Upper Maastrichtian of North America) *Supplement to the Journal of Vertebrate Paleontology* **2013** Annual Meeting Abstract Volume, 159 (2013).

[b61] Bakker R. T., Williams M. & Currie P. J. *Nanotyrannus*, a new genus of pygmy tyrannosaur, from the latest Cretaceous of Montana. Hunteria 1, 1–30 (1988).

[b62] Currie P. J., Hurum J. H. & Sabath K. Skull structure and evolution in tyrannosaurid dinosaurs. Acta Palaeontologica Polonica 48, 227–234 (2003).

[b63] Carr T. D. Craniofacial ontogeny in tyrannosauridae (Dinosauria, Coelurosauria). Journal of Vertebrate Paleontology 19, 497–520 (1999).

[b64] Carr T. D. & Williamson T. E. Diversity of late Maastrichtian Tyrannosauridae (Dinosauria: Theropoda) from western North America. Zoological Journal of the Linnean Society 142, 479–523 (2004).

[b65] Trivedi B., *Tiny tyrant—fossil may be mini* T. rex *cousin*. *National geographic today*. (2002) Avalible at: news.nationalgeographic.com/news/2002/08/0809_0208080_TVhadrosaur.html. (Accessed: 7^th^ December 2015).

[b66] Bakker R. T. & Bir G. 14. Dinosaur crime scene investigations: theropod behavior at Como Bluff, Wyoming, and the evolution of birdness In Feathered dragons: studies on the transition from dinosaurs to birds (eds Currie P. *et al.*) 301–342 (2004).

